# Entropy-Based Effect Evaluation of Delineators in Tunnels on Drivers’ Gaze Behavior

**DOI:** 10.3390/e22010113

**Published:** 2020-01-17

**Authors:** Xueyan Han, Yang Shao, Shaowei Yang, Peng Yu

**Affiliations:** 1 Transportation Technology Building, Chang’an University, Xi’an 710064, China; 2014021039@chd.edu.cn (Y.S.); g106@chd.edu.cn (S.Y.); 2 Northwest Branch of China Airport Construction Group Corporation, Xi’an 710075, China; yu764592985@163.com

**Keywords:** tunnel safety, delineator post configurations, entropy, gaze behavior, driving fatigue

## Abstract

Driving safety in tunnels has always been an issue of great concern. Establishing delineators to improve drivers’ instantaneous cognition of the surrounding environment in tunnels can effectively enhance driver safety. Through a simulation study, this paper explored how delineators affect drivers’ gaze behavior (including fixation and scanpath) in tunnels. In addition to analyzing typical parameters, such as fixation position and fixation duration in areas of interest (AOIs), by modeling drivers’ switching process as Markov chains and calculating Shannon’s entropy of the fit Markov model, this paper quantified the complexity of individual switching patterns between AOIs under different delineator configurations and with different road alignments. A total of 25 subjects participated in this research. The results show that setting delineators in tunnels can attract drivers’ attention and make them focus on the pavement. When driving in tunnels equipped with delineators, especially tunnels with both wall delineators and pavement delineators, the participants exhibited a smaller transition entropy $H_{t}$ and stationary entropy $H_{s}$, which can greatly reduce drivers’ visual fatigue. Compared with left curve and right curve, participants obtained higher $H_{t}$ and $H_{s}$ values in the straight section.

## 1. Introduction

Drivers’ safety on the road is always the most important issue in road design, especially in special sections such as tunnels. Compared with open roads, tunnel environments are poorly lit, the internal space is limited and the driving environment is monotonous and repetitive. The idea that traffic accidents are caused by sudden environmental changes when drivers drive in tunnels was widely discussed [1].

Due to the special environments of tunnels, drivers need to concentrate on the surrounding environment to extract valid traffic information [2]. Mäntyjärvi stated that the information obtained through vision accounts for approximately 80% of the total required information obtained by drivers during driving, of which 95% is dynamic information [3]. However, in many tunnels, drivers can only identify their surrounding environment under low illumination and given monotonous tunnel walls; furthermore, it is difficult for drivers to obtain more effective information visually. Meanwhile, because of the lack of appropriate visual stimulation, drivers tend to feel fatigue and distraction [4].

To improve driving safety in tunnels, many studies enhanced the illumination of tunnels to improve visibility in the tunnel [5,6]. However, improving the lighting conditions in the tunnel alone cannot solve the problem of visual fatigue caused by monotonous tunnel environments [7]. Setting visual guidance facilities can effectively guide drivers’ sight and provide them with appropriate visual stimuli to avoid visual fatigue. As an economical and effective visual guiding device, delineators were used on the road. Similar to the main objective for setting lamps in tunnels, equipping tunnels with delineators can also help drivers be aware of the existence and movement of objects in tunnels [8].

Drivers’ visual characteristics are closely related to safe driving in tunnels [9,10]. As a key modality, the characteristics of vision in tunnels were studied by many scholars. Based on the drivers’ visual adaptation, and considering equivalent veiling luminance, atmosphere luminance and windshield luminance, Mehri et al. designed the required lighting in threshold zone, entrance zone and exit zone of one of long tunnels in Ilam province. By comparing the designed values with the estimated values in the selected tunnel of this study, they concluded that the lighting system of this tunnel was not standard [11]. Through a simulator study, Kircher and Ahlstrom studied the effects of illumination in tunnels on attentive and visually distracted drivers. In the experiment, three levels of illumination were investigated in combination with light-coloured versus dark tunnel walls and attentive versus visually distracted drivers. They found that when driving in tunnels, a driver’s visual attention given to the driving task could significantly affect their visual behavior. Meanwhile, compared with strong illumination, the light-coloured tunnel walls were more important to keep driver’s visual attention focused forward [12]. In other studies, eye movement data were collected through experiments, and basic eye movement parameters were analyzed [13,14]. For example, to investigate how the route familiarity affected drivers’ eye movement features (fixation and saccade) when driving in the entrance zone of highway tunnels with different spatial visual conditions. Hu et al. used eye tracker to record the eye movement data of the drivers at the entrance of the tunnel. By analyzing the variations in the eye movement features, they found that the driver’s familiarity with the road could reduce the adaptation time required by the driver’s eyes when driving through a tunnel [15]. Through an eye movement tracking experiment, Underwood et al. investigated whether the differences in the scanpaths could be associated with the skill acquisition. During the experiment, eye fixations were recorded while the novice and the experienced drivers drove along three types of roads (rural, suburban and dual-carriageway). When the content of fixations were analysed, they identified the single-fixation, the two-fixation, and the three-fixation patterns of eye-movements, and they found that the fixation sequences were different between the novice and the experienced drivers. The experienced drivers showed greater sensitivity and the novices showed some stereotypical transitions in the visual attention [16]. Yan et al. studied how the drivers’ visual characteristics changed as they were passing tunnels. By using eye movement tracking devices, they recorded participants’ test data at tunnel entrance and inside sections. Then they established a relationship model between fixation duration and tunnel distance. Finally, they found that at 100 meters before the entrance of tunnel, the average fixation duration increased. After 100 meters into the tunnel, the fixation duration started to decrease first and then increased [17]. It can be concluded that the analysis of driver visual characteristics is mainly focused on fixations.

Eye movement is an alternating process of fixations and saccades. To study fixation patterns, researchers collected eye movement parameters (such as fixation duration and fixation position) through eye tracking technology [13,14]. For scanpaths, which can be represented by an ordered sequence of fixations [18,19], many studies modeled the ordered sequence as a Markov chain to quantify dynamic eye movement patterns [20]. By modeling the gaze transition as a gaze information channel, Hao et al. calculated the gaze entropy and mutual information to quantify the cognitive comprehension of poster reading [21]. Krejtz et al. calculated the transition entropy and stationary entropy when participants observed paintings from three classical periods and found that individuals’ visual attention switching behavior was related to their personal traits, interests, and recognition of stylized artwork [22]. Gaze entropy can also be used as a reliable surgical task load index, and increasing gaze entropy represents increased task complexity [23,24]. In the transportation domain, gaze entropy is also widely used to evaluate visual exploration features. For example, in Mu et al.’ study, the spectrum entropy, approximate entropy, sample entropy and fuzzy entropy were used to evaluate fatigue driving states. The results showed that the entropy method could achieve good classification performance in driving fatigue studies [25]. Jeong et al. stated that compared with typical static measures, the Markov-based entropy method can quantify eye movement and provide a better understanding of individual switching patterns [26].

This paper mainly studies how different delineator configurations affect drivers’ gaze behavior, which includes fixation and scanpath, in tunnels. Previous studies on delineators were mostly in terms of their influence on driving speed [27,28]. In this study, in addition to studying typical eye movement parameters in a traditional manner, we also quantify the complexity of individual switching patterns using the Markov-based entropy method. According to the previous studies and the actual driving situation in China, this paper assumes that driver focus on the pavement is a positive outcome for safety. Klauer et al. stated that long glances away from roadway were detrimental to safety [29]. Based on Klauer et al.’s conclusion, Kircher and Ahlstrom used “The number of glances away from the road exceeding 2 s” as one of performance indicators to discuss drivers’ visual behaviour, and they stated that an increase of such glances indicated a risk [12]. Thus we think that drivers focus on roadway is a positive outcome for safety. In China, due to the aggravation of traffic congestion, many roads in many cities entered the period of reconstruction and expansion in recent years [30]. When driving on the road, driver’s vision is always blocked by the vehicle in front of them, in rare cases, they can look far away [31,32]. In addition, Huang found that in a tunnel, the driver’s fixation position was 0.604–2.557 s in front of the vehicle, and the distribution of fixation point at night was closer to the front of the vehicle than in the day [33]. Based on the studies above, we assumes that it is positive for safety, if driver spend more gaze time on the pavement.

Based on the actual alignment of the Qinling Mountain No. 1, No. 2 and No. 3 tunnels of the G5 Expressway in Xi’an City (Shaanxi Province, China), this paper designs a simulation experiment that can minimize the interference of external factors and increase the value of the viewpoint information extracted for this study. A total of 25 subjects participated in this research; one drive drove too quickly during the trials, and the eye tracker could not obtain the fixation for three other participants. Finally, data on 21 subjects’ eye movements were used in this study.

The remainder of this manuscript is organized as follows: Section 2 introduces the basic information about gaze entropy. Section 3 presents the experimental details and introduces the data collection. Section 4 mainly analyzes the extracted eye movement data with the traditional method and with the entropy method. Section 5 shows the results of the analysis. Finally, the key findings of the study are summarized in Section 6.

## 2. Gaze Entropy

The concept of entropy was defined by Shannon [34]. Let *X* be a discrete random variable with a probability mass function p(x). The entropy of *X* is defined by

(1) $$H\left(X\right)=-\sum_{x}p\left(x\right)log_{2}p\left(x\right)$$

We use base-2 logarithms, and the entropy is expressed in bits. In information theory, entropy is a measure of uncertainty in a random variable.

Through a discussion of the drivers’ scanpaths, this paper evaluates the effect of different delineator post configurations on drivers’ switching patterns. Thus, to obtain quantitative scanpath comparison metrics, a Markov chain was applied to establish a stochastic model [22].

The Markov chain is a stochastic process with the Markov property in probability theory and mathematical statistics and exists in a discrete index set and state space. For a set of random variables $X=\{X_{t},t>0\}$, if the values of the random variables are all in the countable set $X=s_{i},s_{i}\in s$ and the conditional probability of the random variables satisfies the relationship

(2) $$p\left\{X{}_{t+1}\left|X_{t},\ldots ,X_{1}\right.\right\}=p\left\{X{}_{t+1}\left|X_{t}\right.\right\}$$

where the *X* is called a Markov chain. Specifically, the random variable in step t+1 is conditionally independent on the remaining random variables after giving the random variable in step *t*, i.e., the Markov property.

The Markov property was tested in modeling content-dependent area of interest (AOI) sequences [22]. For each participant, a random sequence $X_{t}$ was used as the scanning process. Let $X_{t}$ take values in the set of AOIs $S=\left\{1,\ldots ,s\right\}$. We can obtain a constant transition probabilities $p_{ij}$ and stationary probabilities $\pi_{i}$, where i,j∈s.

According to previous studies [35,36,37], the scanning process, which is modeled as a Markov process, can be measured by Shannon’s entropy:

(3) $$H_{t}=-\sum_{i\in S}\pi_{i}\sum_{j\in S}p_{ij}log_{2}p_{ij}$$

where $p_{ij}$ represents the empirical probability that the current fixation point is in the $i^{th}$ AOI and that the next fixation point is in the $j^{th}$ AOI, $\pi_{i}$ is the frequency of visits of each AOI, and $H_{t}$ means the average uncertainty of the participant’s gaze transition between AOIs, called the transition entropy, where a higher $H_{t}$ means a higher frequency of conversion between different interest areas [22].

The entropy of the stationary distribution is

(4) $$H_{s}=-\sum_{i\in S}\pi_{i}log_{2}\pi_{i}$$

This represents the average uncertainty of the viewpoint position between different AOIs, called the stationary entropy, where a higher $H_{s}$ indicates that the participant visits more AOIs, whereas lower $H_{s}$ indicates that the participant generally focused on only certain AOIs [21].

## 3. Experiment and Data Collection

### 3.1. Participants

A total of 25 drivers (8 women and 17 men) participated in the study. Their ages range from 23 to 38 years (mean = 29 years, standard deviation = 4.51 years). The participants’ driving experience ranged from 4 years (4 participants) to 5 years (4 participants), 7 years (10 participants), 9 years (5 participants) and 12 years (2 participants), and the total distance driven varied between 2000 km and 300,000 km. The study was approved by the Institutional Review Board of School of Highway, Chang’an University and all participants signed forms indicating informed consent.

### 3.2. Apparatus

The UC-win/road Drive Simulator (Forum 8, Tokyo, Japan) was used in the experiment. Through the combination of virtual reality technology and cockpit, this simulator simulates driving and can realistically simulate driving in a three-dimensional scene. The SMI ETGTM eye tracker (Sensomotoric Instruments, Teltow, Germany), which consists of a head-mounted eye movement instrument, a computer workstation, a mobile recording hard disk and a built-in radio system, was used to extract eye movement data. The BeGaze 3.7 software package (Sensomotoric Instruments, Teltow, Germany) was used for data analysis.

### 3.3. Scenarios

**Figure 1:**
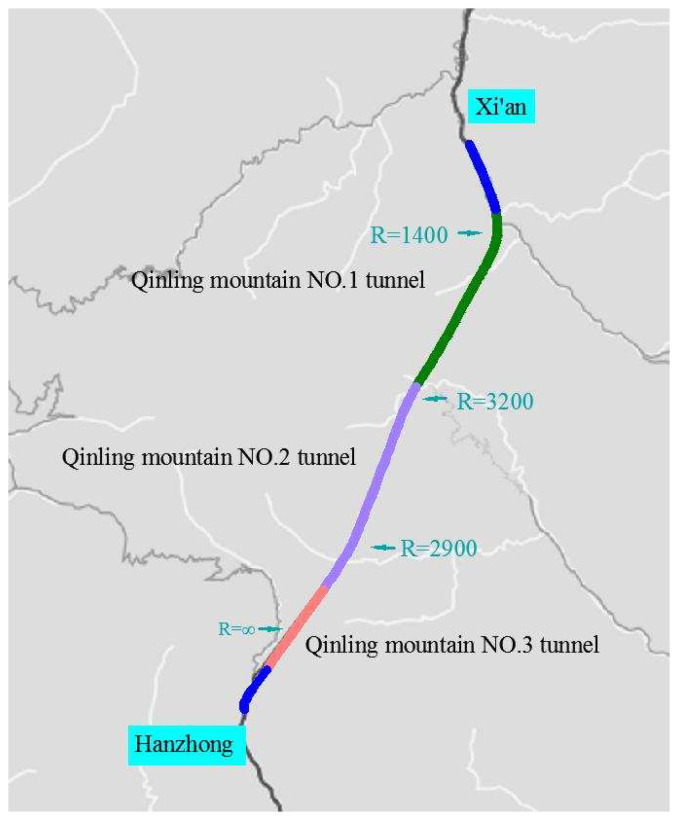
Test route alignment design (Qinling Mountain No. 1, No. 2 and No. 3 tunnels of the G5 Expressway).

The Qinling Mountain No. 1, No. 2 and No. 3 tunnels of the G5 Expressway are located in Xi’an City (Shaanxi Province, China). The expressway is a two-lane road with single-tube tunnels. The total length of the three connected tunnels is 18 km. As shown in Figure 1, the driving direction is from Hanzhong to Xi’an. Within the tunnel section, there are three curves (one right and two left curves). The overall road width is 8.5 m, the lane width is 3.75 m, the left shoulder width is 0.5 m and the right shoulder width is 0.5 m. The posted speed limit is 80 km/h. To explore how delineators affect drivers’ gaze behavior in a tunnel, three different delineator post configurations were designed based on the actual alignments of the Qinling Mountain No. 1, No. 2 and No. 3 tunnels.

**Figure 2:**
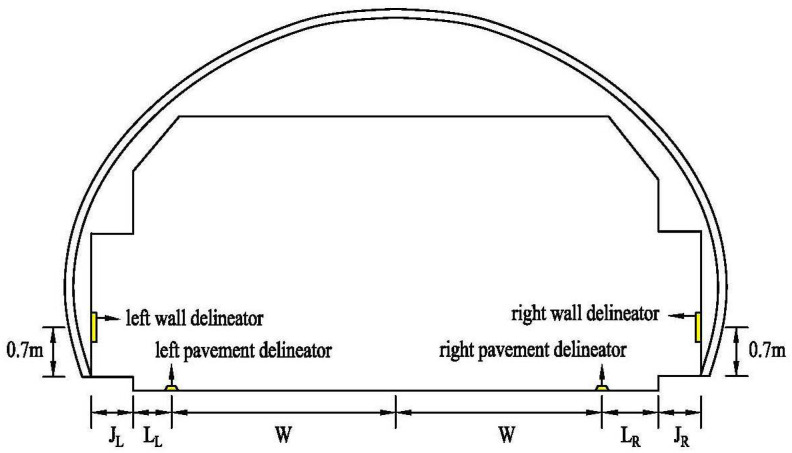
The location of delineators in tunnel.

**Figure 3:**
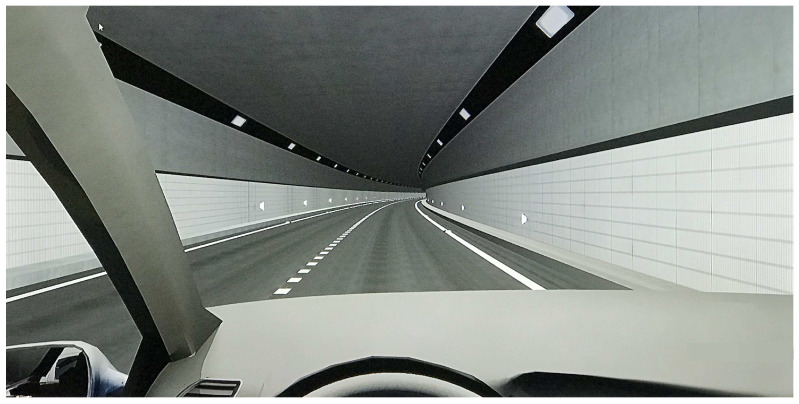
Simulation view in the simulator. This picture is the simulation shown on the middle liquid crystal display when participants drove in scenario A.

The tunnel was equipped with three different delineator post configurations. In scenario A, both the wall delineators and pavement delineators were set. In scenario B, only the pavement delineators were set. In scenario C, there were no delineators. The wall delineators were placed 7 m [38] above the pavement on the side wall, and pavement delineators were placed on the edge marking. Figure 2 shows the location of the delineators. The spacing was 18 m between the right wall delineators, 20 m between the left wall delineators, 21 m between the right pavement delineators, and 22 m between the left pavement delineators [39]. The effect under the driving simulator when participants drove along a right curve in scenario A is shown in Figure 3.

### 3.4. Task and Procedures

There were three delineator settings (both wall delineator and pavement delineator, pavement delineator only and no delineator) combined with three tunnel alignments (straight, left curve and right curve). The two left curve radii were 2900 m and 1400 m, and the right curve radius was 3200 m. To best ensure the comparability of the data in the curve sections, data analysis was applied to the left curve with radius of 2900 m and the right curve with radius of 3200 m. Before the experiment started, the participants put on the eye tracker. They were asked to adjust their body to a comfortable sitting position and do not move their heads too much during the experiment. Then, they were informed of the design of the road and performed a 5-min driving test in the simulator to familiarize themselves with the operating system. In addition, the participants were instructed to drive as usual, and the speed limit was 80 km/h(the driving speed of the subjects was between 75 km/h and 80 km/h, which is close to the speed limit, so this paper does not consider the influence of speed).

### 3.5. Variables

Using the fixation start (ms), fixation duration (ms), fixation end (ms) and fixation position parameters, this paper studied the participants’ gaze behavior in different delineator configurations combined with different alignments.

### 3.6. Data Analysis

In this study, a 3 × 3 (scenario design × tunnel alignment) analysis was used. Of the 25 participants, one drove too quickly during the trials, and the eye tracker could not obtain fixations for the three other participants. Therefore, the useful data came from the remaining 21 participants. The eye movement data in each trial were recorded automatically by the SMI ETGTM eye tracker and analyzed using BeGaze 3.5. Eye movement is an alternating process of fixations and saccades. Using the fixation start (ms), fixation duration (ms), fixation end (ms) and fixation position parameters, we studied the participants’ fixations in different delineator configurations. To explore the scanpath characteristics, we modeled eye tracking fixation sequences between content-dependent AOIs as a Markov chain and calculated every participant’s transition entropy $H_{t}$ and stationary entropy $H_{s}$ to quantify variability of participants’ scan paths between different areas.

## 4. Analysis and Results

### 4.1. Fixation Analysis in Traditional Way

**Figure 4:**
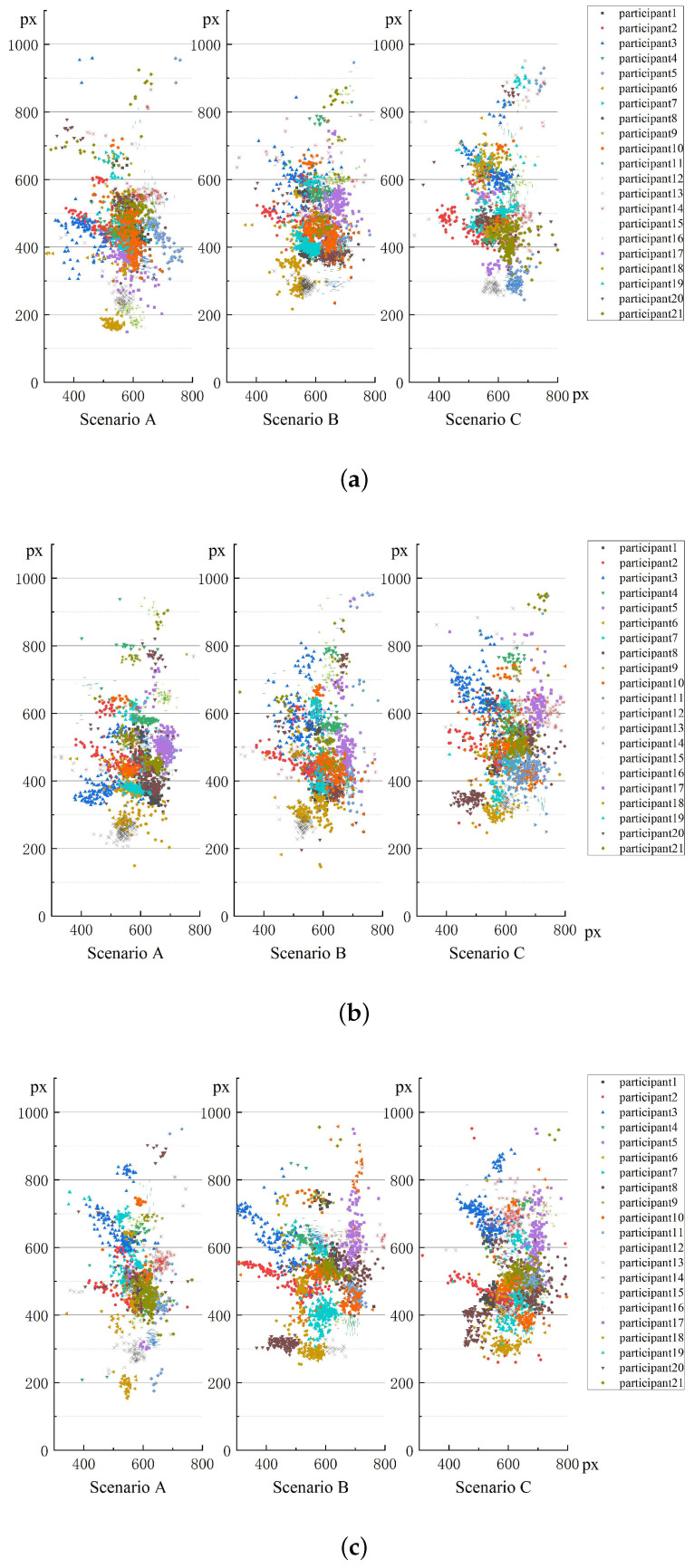
(**a**) Straight; (**b**) Left curve; (**c**) Right curve. Participants’ fixation positions in three different scenarios (In scenario A, both the wall delineators and pavement delineators were set. In scenario B, only the pavement delineators were set. In scenario C, there were no delineators.) combined with three tunnel alignments (straight, left curve and right curve).

Figure 4 shows 21 participants’ fixation positions under three different alignments. As shown in Figure 4, there seems to be a small relative difference between A and C here on the straight road and left curve (as well as between A and B, in fact), in that fixations in C are more upwards.

**Figure 5:**
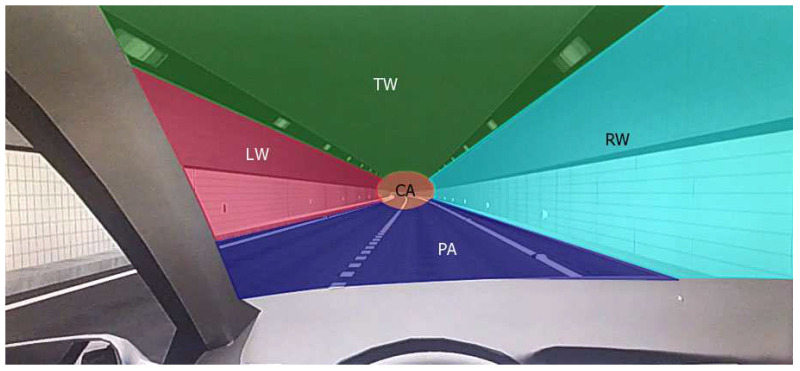
Participant’s visual area division.

Each viewpoint position had a fixation duration. To explore participants’ gaze behavior, first, as shown in Figure 5, we divided the participants’ visual areas into five parts: the top wall (TW), the left wall (LW), the right wall (RW), the central area (CA) and the pavement area (PA). TW included lamps, LW and RW included wall delineators and PA included pavement delineators. The remainder of the driver’s visual range outside of these five areas was defined as white space (WS).

We counted the fixation duration of the 21 participants in these six areas. The percentage of each participant’s fixation duration in each area to the total fixation duration was calculated as follows:

(5) $$T_{s}=\sum_{i=1}^{n}t_{i}$$

(6) $$\beta_{s}=\frac{T_{s}}{T_{total}}$$

where $t_{i}$ means the $i^{th}$ fixation duration in area *s*, s=(1,…,6), $T_{s}$ means the total fixation duration in area *s*, $T_{total}$ is the total fixation duration in these six areas, and $\beta_{s}$ gives the proportion of fixation duration in area *s* to the total fixation duration.

**Figure 6:**
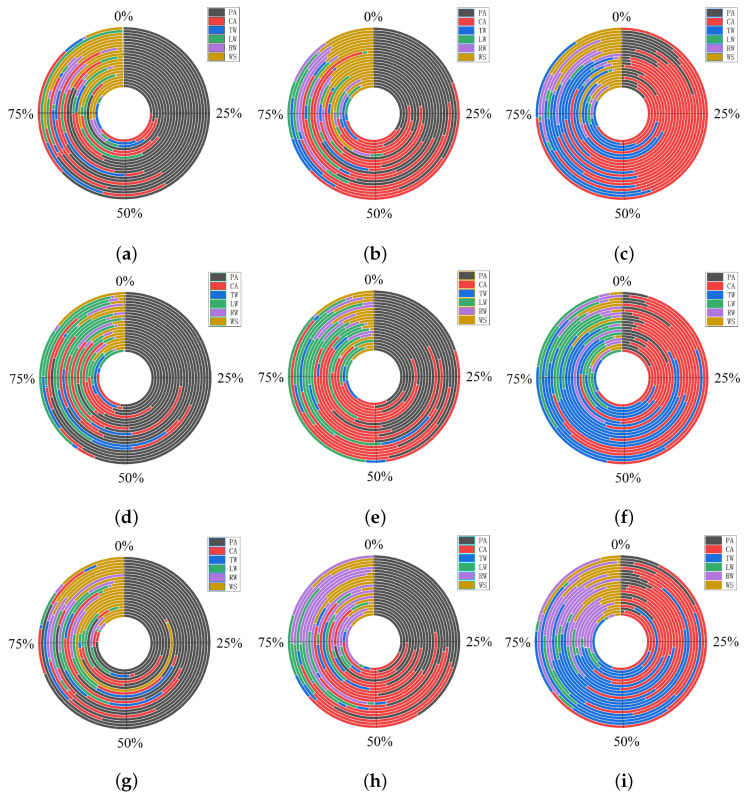
(**a**) straight line in scenario A; (**b**) straight line in scenario B; (**c**) straight line in scenario C; (**d**) left curve in scenario A; (**e**) left curve in scenario B; (**f**) left curve in scenario C; (**g**) right curve in scenario A; (**h**) right curve in scenario B; (**i**) right curve in scenario C. The percentage of fixation duration in three different scenarios (In scenario A, both the wall delineators and pavement delineators were set. In scenario B, only the pavement delineators were set. In scenario C, there was no delineator.) combined with three tunnel alignments (straight, left curve and right curve) for the 21 participants. There are 9 subgraphs, in each subgraph, there are 21 concentric circles. Each concentric circle represents one participant. The six different colors in each concentric circle represent the fixation duration percentage of participant for six different areas, the black, red, blue, green, purple, and yellow parts represent the proportion of gaze time spent in the pavement area (PA), the central area (CA), the top wall (TW), the left wall (LW), the right wall (RW) and the white space (WS), respectively.

The result is shown in Figure 6. There are 9 subgraphs, in each subgraph, there are 21 concentric circles. Each concentric circle represents one participant. The six different colors in each concentric circle represent the fixation duration percentage of participant for six different areas, the black, red, blue, green, purple, and yellow parts represent the proportion of gaze time spent in the pavement area (PA), central area (CA), top wall (TW), left wall (LW), right wall (RW) and white space (WS), respectively.

From Figure 6, we can find that when driving in different scenarios, participants’ interest areas were different. When driving in scenario A, the majority of participants preferred the pavement area (Figure 6a,d,g). In the straight line section and in both the left curve and right curve sections, most of the participants spent more than 50% of their gaze time on the pavement area. While driving in scenario B, the participants mainly focused on the pavement area and central area. As shown in Figure 6b,e,h, the percentage of fixation duration in each area was more balanced than in scenario A. In the straight section, the 21 participants’ fixation duration percentage on the pavement area varied between 20% and 70%; in the central area, the percentage varied from 10% to 60% (Figure 6b). For the left curve, the percentage on the pavement area was from 15% to 65%, and the percentage for the central area was from 15% to 60% (Figure 6e). For the right curve, the percentage on the pavement area varied from 20% to 80%, and the percentage on the central area varied from 10% to 45% (Figure 6h). From Figure 6c,f,i, we can see that participants paid greater attention to the central area and top wall when they drove in scenario C. Driving along the straight line, the percentage varied from 20% to 80% on the central area and from 10% to 65% on the top wall (Figure 6c) area. For the left curve, the percentage on the central area varied from 10% to 70%, and the percentage on the top wall varied from 10% to 80% (Figure 6f). For the right curve, the percentage on the central area varied from 10% to 60%, and the percentage on the top wall varied from 10% to 70% (Figure 6i). Simultaneously, we found that participants liked to focus on the left wall when they drove on the left curve and focused on the right wall when they drove on the right curve. From the results above, we can easily find that under these three scenarios, the driver’s view position moved gradually from the tunnel pavement to the top of the wall. This is consistent with the result shown in Figure 4.

It can be concluded that setting delineators can make a participant focus on the pavement, especially when using both wall delineators and pavement delineators. This may be because the combination of wall delineators and pavement delineators can clearly indicate the edges of the roadway and the forward roadway. When only pavement delineators were set, some drivers identified the direction of the road by the pavement delineators, and others identified the direction by the lamps at the top of the tunnel. However, when no delineators are used, most drivers tend to identify the direction of the road according to the tunnel lamps. Because long glances away from the road are adverse to driver safety [40], we can conclude that the presence of delineators can increase driving safety in tunnels.

### 4.2. Scanpath Analysis Based on Entropy

To further explore the influence of delineator setting on driver gaze behavior under different alignments, the entropy method mentioned above is used to quantify participants’ scanpaths between different areas. The computing process is as follows:

- Collect participants’ eye movement data, including fixation start (ms), fixation duration (ms), fixation end (ms) and fixation position.
- Assign each fixation to AOI sequences. In this paper, the AOIs are $S=\left\{s_{1}\left(PA\right),s_{1}\left(RW\right),s_{1}\left(LW\right),s_{1}\left(TW\right),s_{1}\left(CA\right)\right\}$. The reason for stimulation to appear in the WS area is to divert the driver’s attention to the dashboard to prevent speeding, which is not related to the effect of delineators on a driver’s gaze characteristics. In this section, only eye movement data between the remaining five areas are studied.
- Calculate the probability transition matrix $P=\left[p_{ij}\right]$ [41] and stationary probabilities $\pi_{i}$ as follows:
  (7) $$P=\left[\begin{array}{ccccc} p_{11} & p_{12} & p_{13} & p_{14} & p_{15}\\ p_{21} & p_{22} & p_{23} & p_{24} & p_{25}\\ p_{31} & p_{32} & p_{33} & p_{34} & p_{35}\\ p_{41} & p_{42} & p_{43} & p_{44} & p_{45}\\ p_{51} & p_{52} & p_{53} & p_{54} & p_{55} \end{array}\right]$$
  (8) $$p_{ij}=\frac{n_{ij}}{\sum_{j}n_{ij}}$$
  (9) $$\pi_{i}=\frac{p_{i}}{\sum_{i}p_{i}}$$
  where $p_{i}$ represents the probability that the fixation point is in the $i^{th}$ AOI, i,j∈S.
- Calculate transition entropy $H_{t}$ and stationary entropy $H_{s}$ according to Equations (3) and (4).

An example is given to calculate the entropy of participant 2 in scenario A driving in a straight line. The result is shown as follows:

(10) $$P=\left[\begin{array}{ccccc} 0.667 & 0.067 & 0.044 & 0.089 & 0.133\\ 0.167 & 0 & 0.5 & 0.167 & 0.167\\ 0.2 & 0.05 & 0.5 & 0.2 & 0.05\\ 0.111 & 0 & 0.133 & 0.667 & 0.089\\ 0.154 & 0.077 & 0 & 0.231 & 0.538 \end{array}\right]$$

(11) $$\pi =(\begin{array}{ccccc} 0.310 & 0.042 & 0.148 & 0.317 & 0.183 \end{array})$$

(12) $$H_{t}=-\sum_{i\in S}\pi_{i}\sum_{j\in S}p_{ij}log_{2}p_{ij}=1.593$$

(13) $$H_{s}=-\sum_{i\in S}\pi_{i}log_{2}\pi_{i}=2.098\eta_{p}^{2}=1$$

The entropy values of 21 participants were shown in the Appendix A Table A1, Table A2, Table A3, Table A4, Table A5 and Table A6. A two-way ANOVA was used to test whether the scenarios, alignments, the interaction of scenarios and alignments had significant effects on the participants’ entropy values. The dependent variables were the transition entropy $H_{t}$ and the stationary entropy $H_{s}$, and the independent variables were the scenarios and alignments. The results showed that the scenarios had a significant effect on the drivers’ transition entropy $H_{t}$ (F(2,180) = 12.252, *P* < 0.001, $\eta_{p}^{2}$ = 0.120) and stationary entropy $H_{s}$ (F(2,180) = 16.556, *P* < 0.001, $\eta_{p}^{2}$ = 0.155), and the alignments also had a significant effect on the drivers’ transition entropy $H_{t}$ (F(2,180)=302.425, *P* < 0.001, $\eta_{p}^{2}$ = 0.771) and stationary entropy $H_{s}$ (F(2,180) = 460.013, *P* < 0.001, $\eta_{p}^{2}$ = 0.836). Meanwhile, there was one significant interaction effect on the drivers’ transition entropy $H_{t}$ (F(4,180) = 2.840, *P* = 0.026, $\eta_{p}^{2}$ = 0.059) and stationary entropy $H_{s}$ (F(4,180) = 3.751, *P* = 0.006, $\eta_{p}^{2}$ = 0.077) for scenarios and alignments.

**Figure 7:**
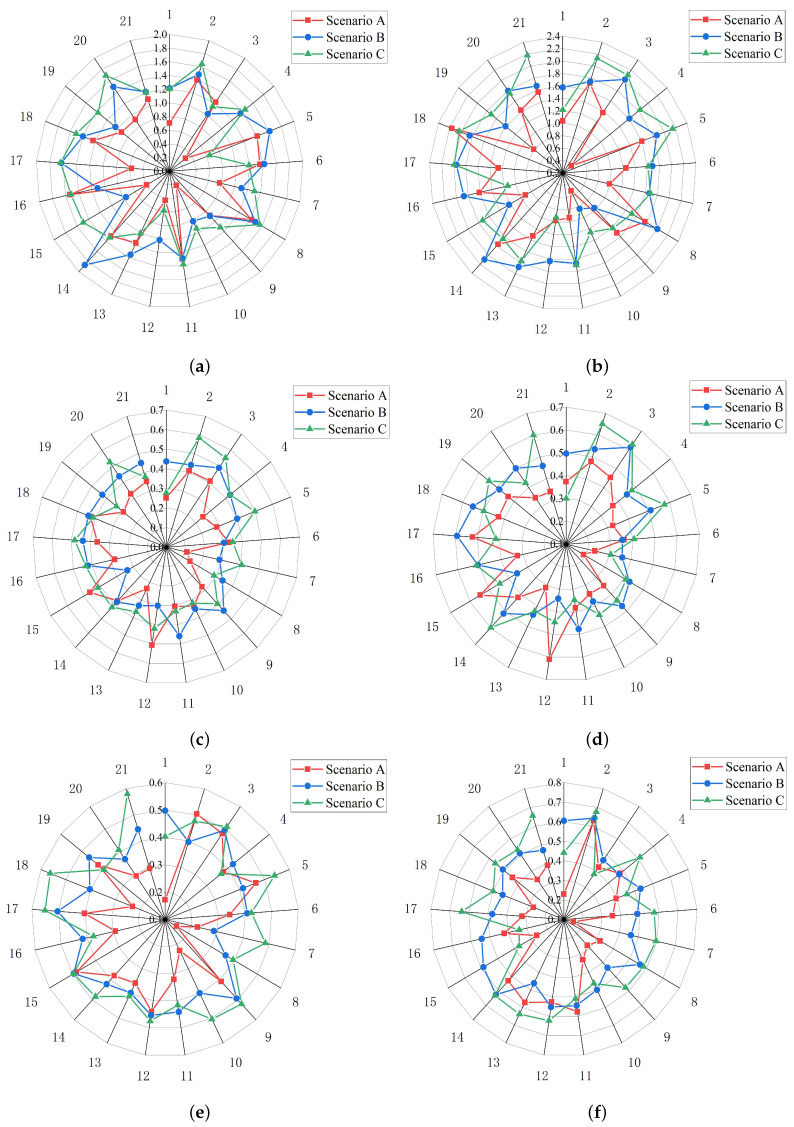
(**a**) $H_{t}$ in straight line; (**b**) $H_{s}$ in straight line; (**c**) $H_{t}$ on left curve; (**d**) $H_{s}$ on left curve; (**e**) $H_{t}$ on right curve; (**f**) $H_{s}$ on right curve. The 21 participants’ entropy values in three different scenarios (In scenario A, both the wall delineators and pavement delineators were set. In scenario B, only the pavement delineators were set. In scenario C, there were no delineators.) combined with three tunnel alignments (straight, left curve and right curve).

The 21 participants’ entropy values in three different scenarios (In scenario A, both the wall delineators and pavement delineators were set. In scenario B, only the pavement delineators were set. In scenario C, there was no delineator.) combined with three tunnel alignments (straight, left curve and right curve) are shown in Figure 7.

In straight line (Figure 7a,b), when participants were driving under scenarios A and B, 86% of them obtained the small transition entropy $H_{t}$ and 90% of them obtained the small stationary entropy $H_{s}$ under scenario A. Compared the entropy values in scenarios A and C, 76% of participants obtained the small transition entropy $H_{t}$ and the small stationary entropy $H_{s}$ under scenario A. When the participants’ entropy values in scenarios B and C were analyzed statistically, approximately two-thirds of the drivers showed smaller $H_{t}$ values (the percentages were 71%) and $H_{s}$ (the percentages were 62%) values in scenario B.

In Left curve (Figure 7c,d), comparing the entropy values in scenarios A and B, 86% of participants obtained the small $H_{t}$ value and the small $H_{s}$ value under scenario A. When participants drove under scenarios A and C, 81% of them obtained the small $H_{t}$ value and 76% obtained the small $H_{s}$ value under scenario A. Analyzing the entropy values in scenarios B and C, 57% of participants obtained the small $H_{t}$ value and the small $H_{s}$ value under scenario B.

In right curve (Figure 7e,f), when participants were driving under scenarios A and B, 90% of them obtained the small $H_{t}$ value and 86% of them obtained the small $H_{s}$ value under scenario A. Comparing the entropy values in scenarios A and C, 86% of participants obtained the small $H_{t}$ value and the small $H_{s}$ value under scenario A. When the participants’ entropy values in scenarios B and C were analyzed, 76% of them obtained the small $H_{t}$ value and 67% of them obtained the small $H_{s}$ value under scenario B.

From the results, it can be found that most drivers’ visual entropy values were the smallest in scenario A, followed by scenario B and scenario C, especially in right curve. Compared with scenario B, when the participants were driving in left curve, only 57% of them showed a higher entropy value in scenario C.

## 5. Discussion

Ni [39] proposed that red-and-white cylinders set at a certain distance from the entrance of a tunnel can be used as a visual reference system for drivers when driving into the tunnel. Similarly, we believe that facilities set up continuously at a certain distance in a tunnel can also be used as a visual reference system for drivers to guide their vision continuously. Therefore, combined with the lamps on the top wall, scenario A provided a three-layer visual reference frame for drivers (one layer for the pavement delineator, one layer for the wall delineator and one layer for the lamp in the top wall). The pavement delineator reference system shows the drivers of the edge marking in the tunnel, and the wall delineator provides the boundary position of the tunnel contour. Most importantly, all three reference systems can show drivers the forward direction of the roadway. Driving under scenario A, participants obtained the minimum $H_{t}$ value. $H_{t}$ indicates the average uncertainty of the participant’s gaze transition between AOIs. The minimum entropy $H_{t}$ in scenario A indicates that when participants drive in scenario A, they have the lowest viewpoint conversion frequency in different AOIs.

Meanwhile, pavement and wall delineators flashing at a certain frequency can continuously give drivers appropriate visual stimulation, and drivers can accurately judge their lateral position from the corner of their eyes; thus, they only need to focus on the road ahead and do not need to change their viewpoints frequently between different AOIs. Whether driving in straight section, left curve or right curve, the minimum $H_{s}$ value in scenario A supports this result because the lower $H_{s}$ value means that participants focuse on certain AOIs [21]. Combined with the analysis of Section 4.1, we find that the participants mainly focus on the PA area under scenario A.

Scenario B was not as good as scenario A in guiding participants’ vision; however, it obtained better effects than scenario C. Scenario B provided a two-layer visual reference frame for participants (one layer for the wall delineator and one layer for the lamp in the top wall). Because of the limited number of pavement delineators, some participants preferred to judge the road direction by the lamps, which made the participants’ $H_{t}$ values larger than that in scenario A. This indicates that the participants’ viewpoint conversion frequency in different AOI is higher than that in scenario A. In Table A4, the value of $H_{s}$ is higher than that in scenario A. This means that under scenario B, participants visited more AOIs, which was consistent with the previous fixation analysis in Section 4.1 (the main AOIs were PA and CA).Compared with scenario A, scenario B was not safe enough.

Analysis of the entropy value in scenario C clearly showed that participants achieved the largest $H_{t}$ and $H_{s}$ values. Scenario C only provided one layer of a visual reference frame (the lamps on the top wall). Fewer visual guidance facilities made participants search for more effective information in tunnels to accurately determine the forward direction on the roadway. Thus, their viewpoint conversion frequency was the highest, and the drivers were more strongly focused on the CA and TW areas. In addition to the above discussion, it seems that compared with linear structures (the edges of the roadway), participants preferred to rely on larger structures (wall delineators, pavement delineators and lamps) to guide their viewpoint in the tunnel. The larger structures improved the visibility of both sides of the road in the tunnel and reduced the drivers’ sense of the distance of the road ahead, while linear structures made the road ahead look far away and monotonous, which readily fatigued the drivers.

Most participants’ visual entropy values ($H_{t}$ and $H_{s}$) were the smallest in scenario A, followed by scenario B and scenario C, especially in right curve. It seems that in right curve, the influence of different scenarios on the participants’ visual entropy values was more significant. Moreover, compared with scenario B, when the participants drove in left curve, only 57% of them showed a higher entropy value in scenario C. We could consider that in left curve, the influence of scenario B and scenario C on the participants’ visual entropy values was not significant enough.

Participants’ visual entropy followed different rules when the drivers drove under different tunnel alignments. From Table A1, Table A2, Table A3, Table A4, Table A5 and Table A6, we find that the participants obtained higher $H_{t}$ and $H_{s}$ values in the straight section. It is not difficult to understand that when driving on a curved section, participants would focus on the inner wall of the curve to determine the tunnel boundary. When they drove on the left curve, the LW area became the main area of focus after the PA, CA and TW areas. When they drove on the right curve, the RW area became the main area of focus after the PA, CA and TW areas. Unlike the curved sections, where the inner wall required the greatest focus, in the straight section, participants’ view positions were more free and arbitrary. Thus, they showed higher $H_{s}$ values (that is, they paid attention to the AOIs that were more balanced) and higher $H_{t}$ values (that is, the viewpoint conversion frequency was higher).

This paper discussed how different delineator configurations affect drivers’ gaze behaviour in different tunnel alignments. Previous studies on delineators mostly centered their influence on the driving speed. In this study, in addition to studying typical eye movement parameters under different scenarios in a traditional manner, we also quantified the complexity of individual switching patterns by using the Markov-based entropy method. However, this experiment was completed in a simulator, we did not consider the influence of the background noise in real-world scenarios, and the different curve radii in tunnels will also influence drivers’ gaze behaviour. In the future, we will improve this aspect of the design.

## 6. Conclusions

This paper reports a simulation experiment to explore how delineators affect drivers’ gaze behavior in tunnels, based on the actual alignment of the Qinling Mountain No. 1, No. 2 and No. 3 tunnels of the G5 Expressway in Xi’an City (Shaanxi Province, China). Through the simulation study, eye movement data on 21 participants were collected. By analyzing these data in a traditional manner, we determined participants’ fixation features. Based on the entropy method, we quantified the participants’ scanpaths between different areas. The conclusions are as follows:

- Compared with the linear structures (the edges of the roadway), drivers preferred to rely on the larger structures (wall delineators, pavement delineators and lamps) to guide their viewpoint in a tunnel. Driving in tunnel equipped with delineators, drivers spent more gaze time on the road ahead and exhibited lower viewpoint conversion frequency in different AOIs.
- Setting delineators can attract drivers’ attention to the pavement. Compared with setting only pavement delineators, setting both wall delineators and pavement delineators can make drivers focus on the road ahead as much as possible.
- Most drivers showed the smallest transition entropy $H_{t}$ and stationary entropy $H_{s}$ in scenario A and the largest $H_{t}$ and $H_{s}$ values in scenario C, especially in right curve. Compared with the left curve and the right curve, participants obtained higher $H_{t}$ and $H_{s}$ values in the straight section.

## Appendix A

**Table A1:** $H_{t}$ values for all participants in straight.

Participants	1	2	3	4	5	6	7	8	9	10	11
Scenario A	0.704	1.393	1.224	0.303	1.409	1.355	0.768	1.416	0.882	0.231	1.294
Scenario B	1.215	1.478	1.016	1.355	1.608	1.422	1.104	1.481	0.889	0.810	1.285
Scenario C	1.204	1.639	1.144	1.444	0.645	1.193	1.304	1.559	1.116	0.928	1.374
**Participants**	**12**	**13**	**14**	**15**	**16**	**17**	**18**	**19**	**20**	**21**	**-**
Scenario A	0.430	1.160	1.290	0.397	1.533	0.571	1.229	0.920	0.912	1.100	-
Scenario B	1.016	1.352	1.867	0.755	1.107	1.626	1.396	1.040	1.494	1.215	-
Scenario C	0.582	1.008	1.320	1.495	1.516	1.637	1.503	1.371	1.693	1.204	-

**Table A2:** $H_{t}$ values for all participants on left curve.

Participants	1	2	3	4	5	6	7	8	9	10	11
Scenario A	0.251	0.407	0.408	0.247	0.283	0.329	0.112	0.144	0.276	0.334	0.306
Scenario B	0.438	0.439	0.490	0.428	0.399	0.303	0.286	0.340	0.442	0.350	0.459
Scenario C	0.277	0.584	0.551	0.428	0.499	0.350	0.405	0.289	0.394	0.317	0.331
**Participants**	**12**	**13**	**14**	**15**	**16**	**17**	**18**	**19**	**20**	**21**	**-**
Scenario A	0.506	0.235	0.374	0.462	0.278	0.362	0.427	0.287	0.329	0.351	-
Scenario B	0.302	0.331	0.381	0.236	0.421	0.439	0.439	0.429	0.438	0.450	-
Scenario C	0.421	0.366	0.418	0.414	0.433	0.481	0.412	0.334	0.525	0.375	-

**Table A3:** $H_{t}$ values for all participants on right curve.

Participants	1	2	3	4	5	6	7	8	9	10	11
Scenario A	0.172	0.503	0.480	0.380	0.465	0.343	0.224	0.149	0.409	0.226	0.321
Scenario B	0.498	0.397	0.495	0.425	0.413	0.408	0.287	0.361	0.493	0.398	0.441
Scenario C	0.403	0.476	0.510	0.368	0.540	0.424	0.485	0.394	0.521	0.504	0.416
**Participants**	**12**	**13**	**14**	**15**	**16**	**17**	**18**	**19**	**20**	**21**	**-**
Scenario A	0.440	0.358	0.380	0.483	0.291	0.402	0.231	0.420	0.292	0.295	-
Scenario B	0.454	0.397	0.422	0.495	0.417	0.504	0.403	0.464	0.367	0.445	-
Scenario C	0.475	0.411	0.484	0.496	0.375	0.551	0.562	0.392	0.408	0.580	-

**Table A4:** $H_{s}$ values for all participants in straight.

Participants	1	2	3	4	5	6	7	8	9	10	11
Scenario A	1.038	1.712	1.376	0.386	1.601	1.246	0.991	1.763	1.512	0.520	0.928
Scenario B	1.577	1.739	2.019	1.605	1.863	1.681	1.663	1.998	0.966	0.839	1.669
Scenario C	1.217	2.130	2.101	1.830	2.144	1.613	1.679	1.515	1.412	1.257	1.696
**Participants**	**12**	**13**	**14**	**15**	**16**	**17**	**18**	**19**	**20**	**21**	**-**
Scenario A	0.971	1.325	1.763	0.910	1.607	1.264	2.163	0.811	1.421	1.561	-
Scenario B	1.632	1.874	2.096	1.222	1.870	1.957	1.849	1.408	1.797	1.664	-
Scenario C	0.919	1.770	1.647	1.723	1.130	1.992	2.032	1.708	1.750	2.177	-

**Table A5:** $H_{s}$ values for all participants on left curve.

Participants	1	2	3	4	5	6	7	8	9	10	11
Scenario A	0.373	0.479	0.453	0.369	0.325	0.360	0.232	0.191	0.348	0.342	0.382
Scenario B	0.497	0.535	0.613	0.449	0.507	0.353	0.358	0.428	0.469	0.378	0.476
Scenario C	0.300	0.651	0.628	0.478	0.574	0.406	0.306	0.410	0.436	0.443	0.347
**Participants**	**12**	**13**	**14**	**15**	**16**	**17**	**18**	**19**	**20**	**21**	**-**
Scenario A	0.609	0.311	0.418	0.546	0.325	0.522	0.426	0.433	0.345	0.340	-
Scenario B	0.341	0.442	0.514	0.355	0.507	0.592	0.550	0.484	0.502	0.459	-
Scenario C	0.445	0.425	0.598	0.445	0.514	0.417	0.497	0.543	0.424	0.600	-

**Table A6:** $H_{s}$ values for all participants on right curve.

Participants	1	2	3	4	5	6	7	8	9	10	11
Scenario A	0.230	0.629	0.423	0.477	0.394	0.356	0.152	0.319	0.281	0.329	0.578
Scenario B	0.605	0.643	0.467	0.471	0.532	0.486	0.460	0.560	0.436	0.499	0.545
Scenario C	0.442	0.676	0.381	0.610	0.455	0.575	0.595	0.582	0.575	0.463	0.509
**Participants**	**12**	**13**	**14**	**15**	**16**	**17**	**18**	**19**	**20**	**21**	**-**
Scenario A	0.527	0.571	0.528	0.264	0.420	0.319	0.271	0.445	0.347	0.390	-
Scenario B	0.553	0.462	0.624	0.587	0.542	0.477	0.445	0.510	0.510	0.471	-
Scenario C	0.623	0.637	0.631	0.371	0.340	0.637	0.498	0.560	0.532	0.655	-
